# The Application and Performance of Artificial Intelligence (AI) Models in the Diagnosis, Classification, and Prediction of Periodontal Diseases: A Systematic Review

**DOI:** 10.3390/diagnostics15243247

**Published:** 2025-12-18

**Authors:** Mohammed Jafer, Wael Ibraheem, Tazeen Dawood, Ali Abbas, Khalid Hakami, Turki Khurayzi, Abdullah J. Hakami, Shahd Alqahtani, Mubarak Aldosari, Khaled Ageely, Sanjeev B Khanagar, Satish Vishwanathaiah, Prabhadevi C. Maganur

**Affiliations:** 1 Department of Preventive Dental Sciences, Jazan University, Jazan 45142, Saudi Arabia; majafar@jazanu.edu.sa (M.J.); wibraheem@jazanu.edu.sa (W.I.); tsararsyed@jazanu.edu.sa (T.D.); 2 Dental Intern, College of Dentistry, Jazan University, Jazan 45142, Saudi Arabia; 202004672@stu.jazanu.edu.sa (A.A.); 202004610@stu.jazanu.edu.sa (K.H.); 3 Department of Periodontics, Riyadh Second Health Cluster, Riyadh 11176, Saudi Arabiamubarkabdullah1393@gmail.com (M.A.); 4 Department of Prosthetic Dental Sciences, King Saud University, Riyadh 11472, Saudi Arabia; abdullah.dentist17@gmail.com; 5 Department of Periodontics, College of Dentistry, Princess Norah bint Abdulrahman University, Riyadh 11671, Saudi Arabia; shahad20alqori@gmail.com; 6 Jazan Health Cluster, Ministry of Health, Jazan 45142, Saudi Arabia; khaledageely1991@gmail.com; 7 Preventive Dental Science Department, College of Dentistry, King Saud bin Abdulaziz University for Health Sciences, Riyadh 11426, Saudi Arabia; khanagars@ksau-hs-edu.sa; 8 King Abdullah International Medical Research Center, Ministry of National Guard Health Affairs, Riyadh 11481, Saudi Arabia

**Keywords:** artificial intelligence, deep learning, classification, diagnosis, grading, panoramic radiographs, periodontal diseases, prediction

## Abstract

**Background/Objectives**: Artificial intelligence is revolutionizing healthcare across multiple areas, and periodontology is no exception to this emerging trend. This systematic study sought to rigorously assess the applicability and efficacy of artificial intelligence (AI) models in the diagnosis, classification, and prediction of periodontal diseases. **Methods**: A web-based search was performed across many reputable databases, including PubMed, Scopus, Embase, Cochrane, Web of Science, Google Scholar, and the Saudi Digital Library. Articles published between January 2000 and January 2025 were included in the search. Following the application of the inclusion criteria, 33 publications were selected for critical analysis utilizing QUADAS-2, and their certainty of evidence was evaluated using the GRADE technique. **Results**: The primary applications of AI technology include the diagnosis, classification, and grading of periodontal diseases; diagnosis of gingivitis; evaluation of the radiographic alveolar bone level and degree of alveolar bone loss; and prediction of periodontal disease risk. The AI models utilized in these studies outperformed current clinical methods in diagnosing, classifying, and predicting periodontal diseases, demonstrating a superior level of precision and accuracy. Their accuracies ranged from 73% to 99.4%, their sensitivities from 75% to 100%, and their precisions from 56% to 99.5%. **Conclusions**: AI has a lot of potential to help with periodontal diagnosis and risk assessment. Its performance is often similar to or better than that of traditional clinical approaches. But before it can be used widely in clinical settings, problems with the quality of the dataset, its generalizability, its interpretability, and its acceptance by regulators must be solved. AI should be seen as a tool that helps doctors make better decisions and not as a way to replace their knowledge and skills.

## 1. Introduction

Periodontal disease, ranked as the sixth most common disease globally [1], is a prolonged inflammatory condition affecting the periodontium [2]. The Global Oral Health Status Report by the WHO in 2022 revealed that severe periodontal diseases impact approximately 19% of the adult population worldwide, which equates to over 1 billion cases [3]. This highly prevalent chronic disease usually starts with the build-up of plaque around the teeth, which then forms microbial biofilms containing bacteria, leading to localized inflammation of the gingiva. Failure to address this can result in the development of chronic periodontal disease [4], characterized by loss of the periodontal ligament and deterioration of the adjacent alveolar bone contributing to tooth loss [5].

Periodontitis impacts people worldwide and can affect individuals of various ages, though it is more commonly seen in older individuals. The higher occurrence and seriousness in this group are a result of the prolonged exposure to known risk factors [6]. Furthermore, a number of illnesses, including peripheral arterial disease, cardiovascular disease, cerebrovascular disease, respiratory diseases, insulin resistance, diabetes, Alzheimer’s disease, respiratory tract infections, and poor pregnancy outcomes, have been found to be associated with this persistent oral infection [7].

Diagnosing periodontitis accurately is challenging for clinicians [8]. The golden standard of identification of the periodontal signs is performed through efficient periodontal charting using a periodontal probe, along with radiographic imaging for assessing the alveolar bone. However, the reliability of these techniques is hindered by variations in the type of periodontal probe, the probing force, the periodontal probing techniques, and the use of different radiographic methods; for example, the position of films could affect the interpretation of radiographs [9,10]. Hence, utilization of AI can help unravel the complexities involved in diagnosing the disease more effectively [1].

The integration of AI in the field of dentistry has been progressively growing over the last decade [11]. Dental professionals and researchers have acknowledged the possibilities that AI offers to improve patient care and simplify clinical processes, like early disease detection and treatment planning by analyzing dental images, thereby improving accuracy and speeding up diagnoses [12]. These tools can also predict patient-specific risks, enabling dentists to implement targeted preventive measures [13].

AI is revolutionizing orthodontics by accurately detecting and classifying malocclusion, thereby enhancing diagnosis, treatment planning, and outcome assessment.

It can also streamline clinical documentation, support remote care, and provide practice guidance [14]. In restorative dentistry, AI helps to detect caries and choose the best excavation method [15], while in endodontics, it can identify root fractures, assess root canals, and predict success rates for retreatment [16]. AI can also be utilized to enhance oral cancer detection by analyzing patient data, like medical history and symptoms, to identify potential risks early on [17]. In addition, AI is assisting dentists in diagnosing temporomandibular joint (TMJ) disorders [18].

AI is still in its early stages of development and has not been extensively utilized in the field of periodontology [19]. Despite the potential benefits of AI in terms of diagnosis and data analysis, there is not enough information to provide a comprehensive overview of its applications in periodontology. Hence, this systematic review was undertaken to assess the application and performance of AI models in the diagnosis, classification, and prediction of periodontal diseases.

## 2. Materials and Methods

### 2.1. Search Strategy

To guarantee the robustness of this systematic review, the authors carefully followed the diagnostic test accuracy criteria specified in the Preferred Reporting Items for Systematic Reviews and Meta-Analyses Extension (PRISMA-DTA) guidelines [20]. According to the PICO (Problem/Patient, Intervention/Indicator, Comparison, and Outcome) paradigm, which is described in Table 1, the search for papers was conducted methodically. The ID record number CRD42024620692 was used to register the protocol for this review with PROSPERO. A computerized search was performed across many reputable databases, including PubMed, Scopus, Embase, Cochrane, Web of Science, Google Scholar, and the Saudi Digital Library, to gather information. Articles published between January 2000 and January 2025 were included in the search. The index terms employed for article searches included artificial intelligence, automated models, artificial neural networks (ANNs), supervised learning, unsupervised learning, machine learning, deep learning, periodontal diseases, gingival diseases, dental plaque, alveolar bone loss, and dental panoramic radiographs for detection, diagnosis, classification, and prediction. We employed Boolean operators (AND, OR) and language filters for English to perform the article search in the electronic databases. Alongside the automated search, we conducted a manual search for pertinent research publications and citations. This involved examining the reference lists of previously obtained papers in the campus library, where physical copies of journals were available. The search was carried out by two independent authors who had been calibrated (S.V. and T.D.).

### 2.2. Study Selection

Two more papers were found by hand search, increasing the original pool to 686 items. The computerized database search yielded 684 articles in total. Based on the substance of their titles and abstracts, as well as their applicability to the study issue, the publications were chosen for additional evaluation. Two people who were not involved in the original search cross-checked every article for duplicates in order to verify that there were no duplicates, which led to the removal of 479 duplicates. After that, 207 full-text papers in all were subjected to a thorough review and data selection process, during which eligibility criteria were used.

### 2.3. Inclusion and Exclusion Criteria

The chosen papers had to satisfy three requirements in order to be considered for inclusion: (a) they had to be original research studies that focused on AI technology; (b) they had to offer measurable values for analysis and assessment; and (c) they had to specify the data that was used to evaluate the AI-based models. Although the study design was unrestricted, this systematic review included only clinical studies. In vivo experimental and animal research was excluded. Additionally eliminated were publications that did not include AI innovation, conference papers or unpublished works that were posted online, articles without full-text copies, and articles written in languages other than English.

### 2.4. Extraction of Data

**Figure 1:**
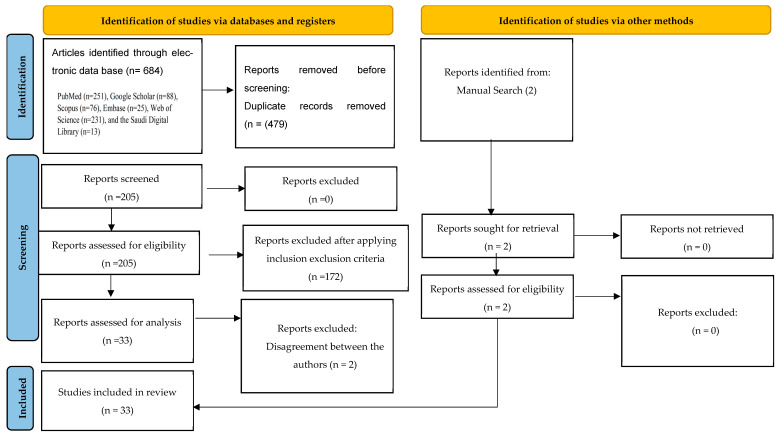
PRISMA 2020 flow diagram for new systematic reviews, which included searches of databases, registers, and other sources.

A total of 35 papers were initially selected for study after the inclusion criteria were applied. Three independent authors who were not involved in the first search (P.C.M., M.J., and S.B.K.) critically evaluated the papers in the second phase after the journal and author data was removed. A Microsoft Excel spreadsheet was created using the data that was taken from the selected articles. This information included the authors, the year of publication, the study’s goals, the kinds of AI algorithms that were utilized, the data sources that were used for the model’s testing, validation, and training, as well as the results, conclusions, and suggestions. However, there were differences among the authors over the inclusion of two publications since there was insufficient evidence to support their results and conclusions. It was determined to exclude them after speaking with the other two authors (S.V. and W.I.). Consequently, as shown in Figure 1, 33 publications were eventually included for qualitative synthesis and were carefully evaluated. The QUADAS-2, which has four categories evaluating different aspects of research design and reporting—patient selection, index test, reference standard, and flow and timing—was used to assess the quality of the included studies [21]. Using Cohen’s kappa on a sample of articles, the two reviewers’ dependability was evaluated, and the results showed an 89% agreement level. Potential sources of bias can be identified, and the findings’ generalizability across various clinical settings and patient groups was assessed by researchers by looking at each area for bias risk and applicability issues.

## 3. Results

Qualitative data was retrieved after 33 articles were thoroughly examined. Over the past ten years, research has shown a growing trend in use of AI for periodontal diagnosis, prognosis, and prediction.

### 3.1. Qualitative Analysis of Included Studies

This systematic review consisted of articles concentrated on four principal categories determined by the commonalities in their fundamental objectives. Each study distinctly outlined a substantial use of AI in clinical practice, such as in the diagnosis, classification, and grading of periodontal diseases [22,23,24,25,26,27,28,29,30,31,32,33], the diagnosis of gingivitis [34,35,36,37], the evaluation of the radiographic alveolar bone level and degree of alveolar bone loss [38,39,40,41,42,43,44,45,46,47,48,49], and the prediction of the periodontal disease risk [50,51,52,53,54], as indicated in Table 2a–d.

### 3.2. Study Characteristics

Details about the authors; year of publication; research objectives; kind of AI model creation algorithm; data sources used for model training, validation, and testing; correctness of the assessment; conclusion; and recommended actions were among the study characteristics taken from the research.

### 3.3. Measures of Outcome

(1) Diagnostic performance—accuracy, sensitivity, specificity, recall, precision, F-1 measure, Positive Predictive Value (PPV), Negative Predictive Value (NPV), and statistical significance; (2) discrimination performance—Receiver Operating Characteristic curve (ROC), Area Under the Curve (AUC), Area Under the Receiver Operating Characteristic (AUROC), mean average precision (mAP), and precision–recall curve (PRC); (3) image basis metrics—Intersection over Union (IoU), Dice similarity coefficient (DSC), and mean absolute error (MAE); (4) reliability/agreement measure—Intraclass Correlation Coefficient (ICC); (5) clinical output measures—radiographic alveolar bone level (RBL) and alveolar bone loss (ABL).

### 3.4. Risk of Bias Assessment and Applicability Concern

As indicated in Table S1, the QUADAS-2 evaluation method was used to evaluate the study’s quality and bias risk. There was low risk of bias in the patient selection domain for both arms because patient data, including dental radiographs and photographic pictures, were used as inputs for the CNNs and ANNs in all included investigations. Because all trials used the same training technique, all arms likewise showed little risk of bias in the index test domain. Furthermore, bias in the flow and timing domain was lessened as a result of AI technology’s usage of standardized methodologies for input data. However, in the risk of bias and applicability arms, there were worries about bias in the index test, reference standard, and flow and timing domains, since two studies failed to provide the reference standard for interpreting index test findings.

**Figure 2:**
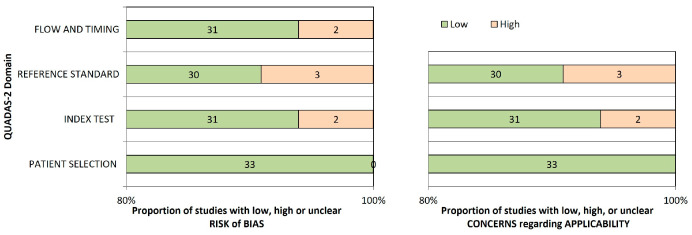
QUADAS- 2 -Assessment of the individual risk of bias domains and applicability.

One of the studies included in the analysis used observations from less experienced dentists as a reference standard. In assessing bias and applicability issues, 10% of the research displayed a significant risk of bias. When all characteristics from the included studies were taken into account, the overall risk of bias in both arms was minimal. The Supplementary Table S1 and Figure 2 provide more details on the risk of bias assessment and applicability issues for the included research.

### 3.5. Assessment of Strength of Evidence

The Grading of Recommendations Assessment Development and Evaluation (GRADE) technique was used to evaluate the degree of evidence certainty in this systematic review [55]. In five domains—risk of bias, inconsistency, indirectness, imprecision, and publication bias—the certainty of evidence was evaluated and classified as very low, low, moderate, or high. Based on this assessment, the studies that were included in this systematic review had a high level of certainty of evidence (Table 3).

## 4. Discussion

Artificial intelligence (AI) has made significant strides in the realm of healthcare, presenting a plethora of exciting innovations and transformative possibilities [55]. Various neural networks and their intricate architectures are employed to efficiently process vast datasets, which particularly helps in medical diagnosis using electronic health records, genomic analyses, and assessments of treatment outcomes to offer valuable information that can assist in diagnosing diseases, tracking patient progress, and optimizing treatment [56]. Periodontal diseases, encompassing gingivitis and periodontitis, persist as a formidable worldwide health issue, owing to their widespread occurrence and systemic complications. Various tests are used to detect periodontal disease, including radiographs, hematological screening, laser therapy, tissue engineering, and more. The conventional diagnostic techniques used currently involve evaluating clinical and radiographic characteristics through instruments and 2D and advanced 3D X-rays [57]. Nevertheless, these techniques have their downsides, with one notable drawback being the lack of uniformity in assessments across different examiners. This inconsistency can lead to disparities in evaluations, potentially affecting the overall reliability and validity of the results obtained. As a result, there is a need for the implementation of AI technology-based tools in order to improve diagnosis and treatment planning [58,59]. With the research available, this systematic review aims to assess the available evidence regarding the application of AI in diagnosing, classifying, and predicting periodontal diseases with the goal of summarizing current practices and guiding future research in this field.

### 4.1. Application of AI for Diagnosing, Classifying, and Grading the Severity of Periodontal Diseases

The early diagnosis, classification, and assessment of the severity of periodontal diseases are very important to determine the most suitable treatment options and enhancement of patient outcomes [4]. Symptoms like swollen gums, easily bleeding gums, persistent bad breath, receding gums, and loose teeth indicate the presence of these diseases, while diagnosis involves a comprehensive clinical examination, measuring pocket depths, and radiographic examination to assess bone loss [60]. These diseases are categorized into different types, including gingivitis (inflammation without bone loss) and periodontitis (inflammation with bone and attachment loss). The severity of periodontitis is determined by stages, ranging from mild bone loss and shallow pockets in Stage I to significant bone destruction and the risk of tooth loss in Stage IV [61]. The disease progression rate is also classified into grades, A (slow), B (moderate), and C (rapid), providing insights into potential tooth loss and guiding treatment choices. Early detection and classification are crucial for treating the periodontal condition effectively, thereby preventing irreversible damage. Leveraging AI tools could significantly decrease the chances of errors due to fatigue or limited expertise in periodontal diagnosis and treatment planning among healthcare professionals [60,61,62].

In a pool of studies [33] reviewed, 13 focused on periodontal disease diagnosis, classification, and severity staging and grading. Ozden F O et al. conducted a notable study where they created a model to classify periodontal diseases accurately based on data from 150 patients and found that Support Vector Machines (SVMs) and decision trees (DTs) were the most effective at categorizing the diseases, achieving a high accuracy rate of 98%, whereas the ANN showed a performance rate of only 46%. In addition, the computational times for SVM and DT were 19.91 and 7.00 s, respectively, indicating that these could serve as valuable diagnostic tools for identifying periodontal diseases accurately, providing support to dental practitioners and helping to minimize errors in interpretation. They even have the potential to identify patients at risk of developing periodontitis and track disease progression. One limitation of the study was the lack of systemic conditions as a risk factor, as the analysis only included clinical and demographic data, limiting the diagnostic sensitivity due to insufficient patient information. Larger, long-term research considering all known risk factors is needed for more accurate diagnoses [24].

In separate research, Papantonopoulos et al. created ANNs capable of categorizing patients with periodontitis into either the aggressive (AgP) or chronic (CP) clinical forms. The ANNs demonstrated a 90–98% accuracy in distinguishing between AgP and CP patients. The most accurate prediction was achieved by an ANN using the absolute counts of monocytes, eosinophils, and neutrophils and the CD4/CD8 ratio as input variables. Thus, ANNs can be utilized for the precise differentiation between AgP and CP based on easily accessible parameters, like peripheral blood leukocyte counts, which would assist clinicians in customizing treatment approaches tailored to the specific needs of patients [25].

Cases of periodontal disease using information from three different parts of electronic dental records (EDRs), including diagnosis codes, clinical notes, and periodontal charting, were identified by Patel J S et al. Through the development of two automated computer algorithms, PD diagnoses were extracted from the EDRs with 100% completeness for 27,138 unique patients for research purposes [31].

A novel deep learning framework was developed by Shon H S et al. to classify the stages of periodontitis in individual teeth using dental panoramic radiographs. Comparing the results of dental specialists, the integrated framework achieved an accuracy of 92.9%, with an average recall and precision of 80.7% and 72.4% across all four stages. The study demonstrated the framework’s high performance, providing valuable support to dental specialists in identifying periodontitis stages for effective treatment [32].

In another investigation by Thanathornwong B et al. [23], only 100 panoramic radiographs were utilized. The quicker R-CNN, trained on a restricted dataset of labeled images, achieved a satisfactory performance in identifying periodontally damaged teeth, attaining a precision of 81%. The utilization of a quicker R-CNN to aid in the identification of periodontally damaged teeth may diminish diagnostic efforts by conserving assessment times and facilitating automated screening documentation.

Across all these studies, a distinct pattern emerged: traditional machine learning models such as SVMs and decision trees exhibited superior performances with clean, structured numerical data, but deep learning techniques thrived alone with extensive, well-annotated imaging datasets. Selecting the appropriate AI model is contingent upon the kind of data it will process, which is essential for practical clinical implementation. Models built on structured variables such as leukocyte levels or CD4/CD8 ratios often outperform deep neural networks when the dataset is small [25]. Meanwhile, convolutional neural networks consistently take the lead in imaging-focused tasks, including radiographic staging [32,44,46,47,48].

### 4.2. Application of AI for Diagnosing Gingivitis

The pre-emptive diagnosis and treatment of gingivitis is crucial, as it can lead to irreversible periodontitis. Alalharith DM et al. demonstrated a Faster R-CNN model for compromised tooth detection with 100% accuracy, precision, and mAP scores, whereas a gingival inflammation detection model achieved a precision of 88.02% and highlighted that deep CNN algorithms were found to have better feature extraction abilities than traditional machine learning methods, resulting in a 10% increase in accuracy compared to conventional techniques. In the same study, the subtle nature of gingivitis, a mild form of periodontal disease, posed challenges in distinguishing inflamed and non-inflamed areas. A 21.5% higher mAP for the non-inflamed class compared to the inflamed class was also noted. The significantly diminished performance of inflamed tissues points out that the first color-based indicators of inflammation are significantly more challenging for CNNs to detect, particularly when the training photos exhibit variations in illumination or camera quality, or when there is little redness. Employing more robust data augmentation or integrating multimodal imaging may enhance accuracy [34].

Li W et al. developed a model using a multitask-learning CNN to screen for gingivitis. By incorporating multitask learning, the model could efficiently handle both classification and localization tasks with a single integrated CNN. Results showed that this model outperformed existing CNNs in accuracy by co-optimizing multiple tasks, leading to improved generalization. The AUCs for detecting gingivitis, dental calculus, and soft deposits were 87.11%, 80.11%, and 78.57%, respectively [37]. Hence, multitask learning can be particularly useful in situations where overlapping visual cues are shared by related disorders, such as calculus, gingivitis, and other deposits. This strategy is becoming progressively more crucial as dental AI develops, assisting the transition to multitarget diagnostic systems [37].

### 4.3. Application of AI to Evaluate Radiographic Alveolar Bone Level and Severity of Alveolar Bone Loss

Alveolar bone loss is a key factor in determining the stage, complexity, prevalence, and distribution of periodontal disease, as per the latest classification of periodontal and peri-implant diseases published in 2017 [63]. In a study by Kurt-Bayrakdar involving 1121 panoramic radiographs, it was found that the AI system utilized showed the highest diagnostic accuracy in identifying total alveolar bone losses (AUC = 0.951) and the lowest in detecting vertical bone losses (AUC = 0.733) [38].

In another study, Jiang L et al. used a CNN model to detect periodontal bone destruction in 640 panoramic radiographs. They identified different bone loss patterns and compared the model’s ability to recognize them with that of dental practitioners. The model showed an accuracy of 77%, outperforming general practitioners, especially in classifying different tooth positions and categories [48].

Kim SH et al. compared five clinicians with AI in detecting bone resorption on 12,179 panoramic radiographs. Clinicians had an average F1 score of 69%, while AI outperformed them with 75%, demonstrating its superior detection of periapical bone lesions compared to dental professionals through a multi-step training framework. This research showed the potential of AI in interpreting radiographs and achieving better results than human clinicians. However, delineating PBL in the third molars (wisdom teeth) remained difficult due to few instances and varied morphologies. Consequently, the model’s accuracy is lower on third molars compared to that of dental professionals [46].

Chang J et al. developed an automatic method to determine bone loss from panoramic radiographs, achieving high accuracy in comparing periodontitis staging with radiologists’ assessments. The automatic method showed better correlations (Pearson correlation coefficient of 0.73) with radiologists than inter-radiologist correlations, indicating its high diagnostic reliability for staging periodontitis [43].

Krois J et al. and colleagues assessed the performances of six dentists and AI using a CNN, with the AL model securing an 81% accuracy rate. The study was more comprehensive, involving multiple dentists, but did not classify PBL patterns as horizontal or vertical. A moderately complex CNN trained on a limited number of images showed a diagnostic performance comparable to that of experienced dentists in PBL detection. The potential use of CNNs to assist dentists in dental imagery diagnostics shows promise [44].

Danks RP et al. [45] employed a deep learning model to ascertain the disease severity stage and the regressive proportion of PBL, surpassing the next most effective architecture by 1.7%. In comparison to doctors’ visual assessments of complete radiographs, the mean PBL error was 10.69%, with an accuracy of 58% in severity staging. This simulates the present inter-observer variability, suggesting that varied data could enhance precision. The technique demonstrated a promising capacity to localize landmarks and assess periodontal bone loss on periapical radiographs.

Kabir et al. developed HYNETS, which integrates multiple segmentation networks and a classification network to provide a comprehensive and accurate solution. It achieved an average Dice coefficient of 0.96 for bone area and tooth segmentation, and an average AUC of 0.97 for periodontitis staging. Results surpassed previous studies, demonstrating a superior performance. Periodontitis classification showed strong agreement with expert evaluation, with no statistically significant differences in radiographic bone loss measurements [47]. This hybrid segmentation model (U-Net, Mask R-CNN) outperformed classification models in bone loss assessment since the former produced more detailed geometric contours of bones, which facilitated the enhancement of the interpretability and clinical relevance [47]. This supports the observation that segmented-based approaches offer great geometric delineation and higher interpretability than traditional classification networks.

CNN-based models were generally successful at identifying moderate to advanced periodontal bone loss, but their performance declined when the disease was in its early stages. This limitation reflects the inherent difficulty of detecting subtle crestal changes or incipient vertical defects on 2D radiographs, where early bone alterations often appear faint or are obscured by anatomical overlap. For example, Kurt-Bayrakdar et al. [38] reported excellent accuracy for generalized bone loss but a markedly lower performance for early or vertical defects. Similar findings were noted by Kim SH et al. [46], where the model performed well overall yet struggled to characterize bone loss around the third molars due to their variable morphology and limited radiographic clarity. These results collectively suggested that while CNNs can detect more pronounced disease reliably, early periodontal breakdown remains a challenge, emphasizing the need for higher-resolution imaging or multimodal approaches to improve early diagnosis.

Lee CT et al. developed a DL-based CAD model that accurately measured alveolar bone levels and provided provisional diagnoses of periodontitis using periapical radiographs, with an accuracy of 85%. Clinicians struggle to determine bone loss stages without manual calculations, emphasizing the value of a CAD tool for assessing radiographic bone loss in clinical decision making. The authors also stated that the bone loss percentage may not always be precise, especially for teeth with short roots or significant tissue attachment. This model might not accurately determine vertical defects’ depth and angulation, crucial for periodontal diagnosis in some cases, and also struggles with identifying missing-teeth numbers accurately. Additional training with more images is needed for a better performance. The authors added that clinical assessment is always necessary for a precise periodontal diagnosis. This DL model also provided data on the CEJ-to-alveolar bone level distance, a unique feature not found in existing models, for clinician reference [39].

Deep learning models consistently perform better when utilizing panoramic radiographs. Models dependent on periapical radiographs, as demonstrated in the study by Lee CT et al. [39], encounter difficulties in identifying vertical abnormalities, variations in root length, and absent teeth. This illustrates the significance of standardized imaging and 3D scans in enhancing AI accuracy.

Shimp N et al. compared five algorithms using clinical data: the Naïve Bayes (NB), Logistic Regression (LR), SVM, ANN, and DT algorithms. The DT and ANN algorithms had higher accuracies in classifying patients for PD risk than the NB, LR, and SVM algorithms. The DT model had 87.08% sensitivity and 93.5% specificity. The radiographic bone level was a significant factor for periodontal disease risk, reinforcing the periodontal disease and type 2 diabetes mellitus association [50]. However, the theradiographic bone level cannot imply the full picture of periodontal risk, and incorporating systemic factors (e.g., diabetes) and lifestyle (smoking) and inflammatory biomarkers expands predictions further.

### 4.4. Application of AI to the Prediction of Periodontal Diseases

Vadzyuk S et al. discovered that psychophysiological features can be effective predictors for detecting the start of gingival disease. Their non-invasive method combines dental exams, index assessments, and psychophysiological indicators to accurately predict gum disease onset. The first model had 83.33% sensitivity and 92.31% specificity, while the second model had 90.00% sensitivity and 78.57% specificity. By using neural network modeling on dental assessments and psychophysiological characteristics, this approach can forecast periodontal disease development in young individuals [51].

Another study [54] found that the deep learning system has predictive accuracies for periodontally compromised teeth (PCT) of 81.0% for premolars and 76.7% for molars. For 64 premolars and 64 molars with clinically confirmed severe PCT, the extraction prediction accuracy was 82.8% for premolars and 73.4% for molars. While periodontists had a higher AUC value, there was no statistically significant difference in predicting accuracy between the two approaches. Lee JH et al. also stated that relying solely on two-dimensional periapical radiographs may not provide a complete diagnosis of periodontal disease. To guarantee accuracy, both radiographic and clinical data must be considered, including the patient’s history and several clinical measures, such as the clinical probing depth, CAL, bleeding on probing, tooth movement, percussion, and electric pulp test. While a deep CNN algorithm with periapical radiographs can help diagnose PCT, it may not be adequate on its own. Implementing a three-dimensional deep CNN algorithm with CT and MRI data shows potential for improved diagnosis and prediction. Many prior deep CNN investigations employed downscaled, low-resolution medical pictures due to practical restrictions, potentially impacting the accuracy. Ongoing improvements in deep learning algorithms are enhancing the diagnosis and prediction accuracy, which will make computer-aided diagnosis a valuable tool in the future [54].

Another pattern observed across the included studies was that models relying solely on 2D radiographs showed only moderate predictive performances, typically ranging between 76 and 82%**,** especially when assessing early periodontal breakdown. This trend is clearly demonstrated in Lee JH et al., where the deep learning system achieved high accuracy for premolars and molars using only periapical radiographs [54]. When additional clinical parameters, such as the probing depth, the CAL, bleeding on probing, mobility, or systemic factors, were incorporated, several models showed marked improvements in diagnostic specificity. Shimp N et al. [50] reported higher classification accuracy when radiographic findings were combined with clinical indicators and diabetes status, and Papantonopoulos et al. [25] achieved high accuracy (90–98%) when leukocyte profiles and CD4/CD8 ratios were included. Collectively, these findings support the growing recognition that multimodal fusion approaches, which integrate radiographic, clinical, and demographic information, offer a more robust foundation for next-generation periodontal AI systems.

Across the 33 studies reviewed [22,23,24,25,26,27,28,29,30,31,32,33,34,35,36,37,38,39,40,41,42,43,44,45,46,47,48,49,50,51,52,53,54], every investigation relied on supervised learning, whether the models were built from radiographs, clinical periodontal measurements, immune biomarkers, electronic dental records, or psychophysiological data. The largest group consisted of radiograph-based supervised CNN models, many of which focused on staging or quantifying bone loss [22,32,34,37,38,39,43,44,46,47,48]. Studies using clinical or other structured variables, such as demographic information, periodontal charting, systemic or immune markers, and risk-factor profiles, constituted another key group [23,24,25,31,50,51]. Several papers also explored predictive or hybrid machine learning approaches [26,27,28,29,30,33,35,36,40,41,42,45,49,52,53,54]. Interestingly, none of the studies employed unsupervised or self-supervised methods, even though these approaches could help reveal hidden patterns in periodontal disease. Transfer learning, typically via pre-trained CNN backbones, was used in multiple imaging studies [34,37,39,46,47,48] and consistently improved the performance in small datasets, a common limitation across nearly all included research. Although a few studies integrated more than one data source, such as combining radiographic, clinical, or systemic inputs [25,31,39,50], fully developed multimodal fusion remains rare in periodontal AI research.

## 5. Advanced AI Frameworks in Periodontal Diagnostics

Most studies in this review focused on conventional deep learning models, which is reasonable because these are the methods most commonly applied to radiographs and periodontal datasets. However, AI in healthcare is moving rapidly, and several developments now extend far beyond image classification. Ignoring these advances would limit the relevance of the discussion, especially given the current interest in clinical decision support systems.

One area that has gained particular attention is Retrieval-Augmented Generation (RAG) [64]. Traditional neural networks operate only on the information they were trained on, which means that they may give incomplete or occasionally incorrect responses when faced with unusual cases. RAG addresses this limitation by allowing the model to retrieve validated external information before generating a prediction or explanation [65]. In a periodontal setting, such a system could examine a radiograph and, at the same time, pull updated AAP case definitions, outcome data from recent regenerative studies, or even patient-specific risk factors, such as diabetes or smoking. The result is not just detection of bone loss but a more informed explanation and potentially a better-reasoned clinical recommendation [64].

Another major milestone is the rise of multimodal foundation models. These include large language models adapted for healthcare, such as GPT-4-based medical systems and Med-PaLM [66]. Unlike typical CNNs, these models can interpret several forms of information at once—for example, radiographs, periodontal charting, photographs, and even microbiome or genetic data. Because all inputs are processed within a unified framework, the model can recognize relationships that are easy to miss when each dataset is analyzed separately. Reports in medical imaging have already shown that multimodal models can describe findings, summarize charts, and explain clinical impressions in a way that resembles human reasoning [67]. Although this technology has not yet been meaningfully applied to periodontics, it represents a major direction for future research.

### 5.1. Model Interpretability and Explainability

For AI to be accepted in clinical dentistry, clinicians must understand why a model arrives at a particular decision. Image-based systems often use methods such as Grad-CAM, which highlights the area of the radiograph that influenced the output, like the crestal bone margin or a furcation defect [68]. For models that combine clinical or demographic variables, tools such as Shapely additive explanation (SHAP) help clarify how each feature contributed to the prediction [69]. These methods make it easier for clinicians to judge whether the output is reasonable, and they are increasingly expected by regulatory bodies that require transparency in algorithmic decision making.

### 5.2. Data Imbalance, Bias, and Ethical Considerations

Despite these promising developments, several limitations remain. Periodontal datasets often contain far more advanced cases than early ones, and demographic diversity is usually limited. When models are trained on such unbalanced data, their performances tend to drop for minority groups or less represented disease stages. Addressing this requires deliberate data collection strategies and the routine use of bias assessment tools. Large multimodal models are also at higher risk of overfitting, especially because dentistry datasets are usually small [70]. External validation across multiple clinics, populations, and imaging systems is therefore essential before these models can be considered reliable. Ethical considerations also need to be taken care of. The responsibility for errors made by an AI system must be clearly defined, and clinicians should avoid over-relying on automated outputs. Systems that combine radiographic, clinical, and personal health information must also provide strong data protection measures because such models expose more sensitive information [71].

### 5.3. Limitations

Even though the benefits of the widespread use of AI in periodontics are well acknowledged, certain challenges and restrictions must be well addressed. A lack of regulatory frameworks, reservations about data privacy, and the need for high-quality datasets are some of these challenges. Cost, infrastructure, and acceptance by practitioners are possible barriers to accepting AI technologies in periodontal therapy. AI systems indeed require large amounts of diverse, high-quality data to be trained effectively. However, it is challenging to collect such data, particularly in regions where access to high-end dental imaging or digital records is limited. Furthermore, lack of diversity within training datasets may result in AI models being unable to generalize across populations, which can lead to bias in the diagnosis and recommendation of treatment [55,72].

The lack of transparency in image detection and classification presents a significant challenge. Neural networks, in their essence, fail to offer a definitive rationale for the specific decisions they arrive at and thus lack the capacity to substantiate the diagnoses issued by a provider, particularly in the field of medicine. It is crucial for navigating regulatory oversight and optimizing the algorithm to enhance its applicability in clinical environments. The endeavor is being undertaken for clinical objectives, aiming to develop AI technologies that facilitate comprehension of AI decision making; nonetheless, a viable solution for this purpose remains elusive. Dental professionals should recognize that artificial intelligence is intended to enhance their practice, serving as an efficient diagnostic tool that complements expert judgment in clinical settings. This level of coherence guarantees optimal patient well-being [55]. The integration of AI tools may necessitate extended training for dental professionals, a scenario that poses challenges for small-scale practices or those with constrained resources [72].

Ensuring the confidentiality and security of information is a major challenge for AI in periodontics since it serves by basing its findings on sensitive health information. Therefore, rigorous compliance with laid-down guidelines in the preservation of confidentiality and an array of regulatory requirements must be maintained. Clinicians relying on AI tools in their practices face ethical dilemmas in maintaining responsibility for errors due to ambiguity as to who is accountable [72]. The prospect of AI technologies seems palpable but may not seem all too feasible financially for smaller practices or in scenarios with limited resources. The cost of AI systems, infrastructure, and training in one way or another could end up creating disparities in access to periodontal care, especially in developing countries. There would arise a need for the provision of access and affordability of AI tools for patients across the board regardless of socio-economic class or geographical setting [72,73,74]. In addition, resistance to change, incorporation of the new technology within existing structures, and maintenance costs remain some of the hurdles in service delivery whenever new technology is introduced. Among the recent critiques, concerns about the presence of implicit biases in the training datasets and their influence on the performance of AI are also mentioned [72,73,74]. In the future, researchers should instead concentrate on assembling carefully selected datasets from several institutions rather than small isolated collections. Testing multimodal and RAG-based systems in actual clinical settings is equally as crucial as testing them in controlled laboratory settings. To ensure that these tools are truly reliable, we also need more precise rules about things like interpretability and fairness [64]. The ultimate objective is to provide clinicians with more intelligent, reliable tools that enable them to diagnose patients more accurately and provide more individualized care.

The implication of machine learning is no longer restricted to analysis of images but is also emerging as an essential tool in identification of the predominant periodontal pathogens that initiate and progress periodontal disease by focusing on the subgingival ecosystem as a whole [75]. Feher et al. found that the presence of subgingival microflora could predict the prognosis of periodontal therapy. Pattern recognition helps clinicians to identify certain subtle pathogens that could be missed by just observing a patient’s baseline microbiome, as the prediction of these specific predominant periodontal pathogens could influence the periodontal treatment outcome [76]. The recognition of a patient’s subgingival micro-organisms prior to therapy can provide a significant roadmap in the management of periodontal disease, highlighting the need for any specific treatment modality that could benefit the overall periodontal success.

Modern sequencing tools now generate huge amounts of data on bacterial levels, interaction patterns, and functional genes that are far too complex to sort manually. This is where AI becomes genuinely useful. Methods such as unsupervised clustering and supervised prediction models can pick up subtle microbial patterns that relate to clinical symptoms, separating healthy and diseased biofilm profiles more accurately than traditional methods [76].

## 6. Conclusions

AI has a lot to offer in terms of early disease identification, proper diagnosis, the adequate prediction of periodontal disease progression, and the monitoring of treatment outcomes; however, the shortcomings include challenges concerning data accuracy, clinical integration, ethical dilemmas, and costs. Therefore, conducting AI’s efficient induction in the field of periodontology will gain leverage by discussing these challenges, investing in research and development, and ensuring that such programs assist dental professionals in their everyday practice. Finding this balance will help AI become part of the large-scale transformation in periodontal care and patient outcomes.

## Figures and Tables

**Table 1:** Description of the PICO (P—Population, I—Intervention, C—Comparison, O—Outcome) elements.

Research question	How well do AI-based models predict, classify, and diagnose periodontal diseases?
Population	Patients who underwent investigation for periodontal diseases, including those assessed using radiographs (periapical, bitewing, panoramic), intraoral images, and periodontal clinical examination.
Intervention	AI models designed for the diagnosis, classification, and prediction of periodontal diseases.
Comparison	Expert/specialist opinions and reference standards/models.
Outcome	Diagnostic, classification, and predictive performance metrics of AI models were predefined and grouped as follows: (1) Diagnostic performance—accuracy, sensitivity, specificity, recall, precision, F1 measure, Positive Predictive Value (PPV), Negative Predictive Value (NPV), and statistical significance; (2) Discrimination performance—Receiver Operating Characteristic curve (ROC), Area Under the Curve (AUC), Area Under the Receiver Operating Characteristic (AUROC), mean average precision (mAP), and precision–recall curve (PRC); (3) Image basis metrics—Intersection over Union (IoU), Dice similarity coefficient (DSC), and mean absolute error (MAE); (4) Reliability/agreement measure—Intraclass Correlation Coefficient (ICC); (5) Clinical output measures—radiographic alveolar bone level (RBL) and alveolar bone loss (ABL).

**Table 2:** Qualitative synthesis of included studies.

**Table 2a. Application of AI for Diagnosing, Classifying, and Grading the Severity of Periodontal Diseases**													
**Sl No.**	**Authors**	**Year of Publication**	**Study Design**	**Algorithm** **Architecture**	**Objective of the Study**	**No. of Patients/Images/Photographs for Testing**	**Primary Objective**	**Modality**	**Comparison, If Any**	**Evaluation Accuracy/Average Accuracy/Statistical Significance**	**Results:** **(+) Effective,** **(−) Noneffective, (N) Neutral**	**Outcomes**	**Author Suggestions/Conclusions**
1	Ossowska A et al. [22]	2022	Retrospective study	ANN	To assess grades of periodontitis based on severity	110 patients: training group: 90 persons; test group: 20 persons.	Severity of periodontitis	Datasets	Training and test group comparison	Sensitivity = 85.7% Specificity = 80.0% Percentage of correctly classified patients = 84.2% for the training set	(+) Effective	ANNs were used correctly to classify patients according to the grade of periodontitis	ANNs may be useful tools in everyday dental practice to assess the risk of periodontitis development.
2	Thanathornwong B et al. [23]	2020	Retrospective study	Faster R CNN	To identify periodontally compromised teeth	100 digital panoramic radiographs	Detection of periodontally compromised teeth	DPRs	Three experienced periodontists	Precision = 81% Recall = 80% Sensitivity = 84% Specificity = 88% F measure = 81%	(+) Effective	Faster R-CNN trained on a limited number of labeled imaging data had satisfactory detection ability of periodontally compromised health	Application of Faster R-CNNs may reduce diagnostic effort by saving assessment time and enabling automated screening documentation.
3	Ozden F O et al. [24]	2015	Retrospective study	SVM DT ANN	To develop an identification unit for classifying periodontal diseases	150 patients divided into two groups [training (100) and testing (50)]	Classification of periodontal diseases	Datasets	Experienced periodontist	Performances of SVM and DT = 98% Performance of ANN = 46% Total computational times of SVM and DT: 19.91 and 7.00 s	(+) Effective	DT and SVM were the best to classify the periodontal diseases. The ANN had the worst correlation between input and output variables.	A unique system for diagnosing periodontal diseases may be possible.
4	Papantonopoulos G et al. [25]	2014	Retrospective study	MLP ANNs	To classify patients into aggressive periodontitis (AP) or chronic periodontitis (CP)	a. First study (29 patients) b. Second study (76 patients) c. Third study (80 patients)	Classification of periodontitis	Datasets	Canonical discriminant analysis and binary logistic regression	ANNs gave 90–98% accuracy in classifying patients as having either AgP or CP	(+) Effective	ANNs can be employed for the accurate diagnosis of Ag P or CP.	ANNs allow clinicians to better adapt specific treatment protocols for their AgP and CP patients.
5	Xiang J et al. [26]	2022	Observational study	Random forest algorithm and ANN	To construct a diagnostic model for periodontitis	Two datasets containing (64 and 183) and (69 and 241) periodontitis samples	Diagnosis of periodontitis	Gene expression data	Not mentioned	AUC = 0.945; ROC = 0.900	(+) Effective	The authors successfully identified key biomarkers of periodontitis using machine learning and developed a satisfactory diagnostic model.	The model provides a valuable reference for the prevention and early detection of periodontitis.
6	Farhadian M et al. [27]	2020	Cross-sectional study	ANN	To automate diagnoses of various periodontal diseases	300 patients: 160: gingivitis; 60: localized periodontitis; 80: generalized periodontitis	Diagnosis of periodontal disease	Datasets	Not mentioned	Overall correct classification accuracy of 88.7%; overall hypervolume under the manifold value of 0.912; and has the best performance	(+) Effective	The designed classification model has an acceptable performance in predicting periodontitis.	This system will help less experienced dentists and young residents in making decisions for the diagnosis of periodontal disease.
7	Chifor R et al. [28]	2022	Observational study	Mask R CNN and U-Net	To identify anatomical elements for periodontal diagnosis	3417 periodontal U.S. images to form the datasets for training	Diagnosis of periodontitis	Ultrasound images	Low-experience operator (young dentist)	IOU is 10% for the periodontal pocket and 75.6% for gingiva	(+) Effective	Mask R-CNN had overall better results in the automatic segmentation of periodontal tissue in ultrasound images, compared with U-NET.	A method like this may help a less experienced operator to generate higher-quality datasets in the future.
8	Arbabi S et al. [29]	2018	Retrospective study	LM SCG	To evaluate the role of ANNs in periodontal disease diagnosis	190 periodontal disease cases [training: 160; testing: 30]	Diagnosis of periodontitis	Datasets	Comparison between two algorithms	LM algorithm’s training in 22 performances gained 0.0098, and the SCG algorithm’s training in 33 performances had 0.055 for the MSE	(+) Effective	The LM algorithm with fewer iterations and a minimum MSE had a better performance than that of the SCG algorithm.	ANNs can be used as an effective tool.
9	Su S et al. [30]	2022	Observational study	Mask R-CNN	To develop a computer-assisted system based on a CNN to segment and calculate the root surface area on CBCT	24 teeth from 20 patients; CBCT images were recorded	Diagnosis of periodontitis	CBCT images	Medical image control system (Mimics)	Mean RSA difference between two groups was −0.20 ± 5.1 mm Alveolar bone mAP = 0.848 ± 0.004; mIOU = 0.715 ± 0.004	(+) Effective	The CNN is an automatic, efficient, standardized, and accurate method to calculate the RSA.	The CNN can help dental professionals attain more targeted subsequent clinical or radiographic diagnostics and treatment on CBCT.
10	Patel JS et al. [31]	2022	Retrospective study	PeriodDx diagnoser and PerioDx extractor	To phenotype periodontal disease diagnoses from different sections	27,138 data points of patients	Diagnosis of periodontal disease	Electronic dental records	Two domain experts	The PerioDx diagnoser performed with 96% precision, 98% recall, and 97% of the F-1 measure. Similarly, the PerioDx extractor performed with 91% precision, 87% recall, and 95% of the F-1 measure to automatically extract patients’ PD diagnoses.	(+) Effective	Successfully developed, tested, and deployed two automated algorithms on big EDR datasets to improve the completeness of PD diagnoses with 100% completeness.	This approach is recommended for use in other large databases for the evaluation of their EDR data quality and for phenotyping PD diagnoses and other relevant variables.
11	Shon, H.S. et al. [32]	2022	Retrospective study	U-Net and YOLOv5	To classify periodontitis stages of each individual tooth using dental panoramic radiographs	1044 images	Classification of periodontitis	DPRs	Dental specialist	The integrated framework had an accuracy of 92.9%, with a recall and precision of 80.7% and 72.4%, respectively, on average, across all four stages.	(+) Effective	The novel framework was shown to exhibit a relatively high level of performance.	A systematic application will be developed in the future to provide ancillary data for diagnosis and basic data for the treatment and prevention of periodontal disease.
12	İçöz D et al. [33]	2023	Observational study	DL models YOLOv3 Darknet model	To evaluate the effectiveness of an artificial intelligence (AI) system in the detection of roots with apical periodontitis (AP)	306 DPRs	Diagnosis of periodontitis	DPRs	Two oral and maxillofacial radiologists	Recall = 98% Precision = 56% F-1 measure = 71%	(+) Effective	The DL method developed for the automatic detection of AP showed high recall, precision, and F-1 measure values for the mandible but low values for the maxilla.	The performance of YOLO can be improved by dimensionally classifying the lesions and by including a sufficient and equal number of training and testing data on the basis of each tooth group.
**Table 2b: Application of AI to diagnose gingivitis**													
**Sl No.**	**Authors**	**Year of publication**	**Study Design**	**Algorithm** **Architecture**	**Objective of the study**	**No. of Patients/Images/Photographs for Testing**	**Primary Objective**	**Modality**	**Comparison, If Any**	**Evaluation Accuracy/Average Accuracy/Statistical Significance**	**Results:** **(+) Effective,** **(−) Noneffective, (N) neutral**	**Outcomes**	**Author Suggestions/Conclusions**
1	Alalharith D.M et al. [34]	2020	Retrospective study	Two faster region-based CNN models using ResNet-50 CNN	To detect and diagnose early signs of gingivitis	134 intraoral images (107 for training and 27 for testing)	Diagnosis of gingivitis	Intraoral image dataset	Expert dentists	Inflammation detection model: Accuracy = 77.12% Precision = 88.02% Recall = 41.75% mAP = 68.19%	(+) Effective	This study proved the viability of deep learning models for the detection and diagnosis of gingivitis in intraoral images.	This model can be used in the field of dentistry and aid in reducing the severity of periodontal disease globally through pre-emptive, non-invasive diagnosis.
2	Li W et al. [35]	2020	Dataset	MGLCM + PSONN)	To automate diagnosis of chronic gingivitis	400 gingivitis and 400 healthy images were acquired to build the training dataset	Diagnosis of gingivitis	Oral images	State-of-the-art approaches	Specificity: 78.1% Sensitivity: 78.2% Precision: 78.2% Accuracy = 78.2% F1 score = 78.1% of MGLCM (PSONN as a classifier) method	(+) Effective	The model is an efficient and accurate method.	Provides new ideas with the application of AI technology to diagnose periodontal disease and help dentists with laborious tasks.
3	Li W et al. [36]	2019	Dataset	CLAHE + GLCM + ELM	To automate diagnosis of chronic gingivitis	93 images; 58 gingivitis and 35 healthy images	Diagnosis of gingivitis	Oral images	Conventional methods	Sensitivity, specificity, precision, and accuracy of our method are 75%, 73%, 74%, and 74%, respectively.	(+) Effective	The models were more accurate and sensitive than state-of-the-art approaches.	The combination of CLAHE, GLCM, and ELM is an efficient and accurate method to classify tooth types and diagnose gingivitis.
4	Li W et al. [37]	2021	Retrospective study	CNN model	To automate screening of gingivitis, dental calculus, and soft deposits	Out of 625 patients, 3932 oral photos were captured [training, validation, and testing subsets]	Diagnosis of gingivitis	Oral photos	Three board-certified dentists	AUC for detecting gingivitis, dental calculus, and soft deposits were 87.11%, 80.11%, and 78.57%, respectively.	(+) Effective	The model significantly outperformed on both classification and localization tasks, which indicates the effectiveness of multitask learning on dental disease detection.	The model could be meaningful for promoting public dental health.
**Table 2c: Application of AI to evaluate radiographic alveolar bone level and severity of alveolar bone loss**													
**Sl No.**	**Authors**	**Year of Publication**	**Study Design**	**Algorithm** **Architecture**	**Objective of the Study**	**No. of Patients/Images/Photographs for Testing**	**Primary Objective**	**Modality**	**Comparison, If Any**	**Evaluation Accuracy/Average Accuracy/Statistical Significance**	**Results:** **(+) Effective,** **(−) Noneffective, (N) Neutral**	**Outcomes**	**Author Suggestions/Conclusions**
1	Kurt-Bayrakdar S et al. [38]	2024	Retrospective study	CNN	To examine the performance of this algorithm in the detection of periodontal bone losses and bone loss patterns	1121 DPRs: training set (80%), validation set (10%), and testing set (10%)	Assessment of alveolar bone loss	DPRs	Three periodontists and one oral maxillofacial radiologist	**ABL** Sensitivity = 100% Precision = 99.5% F1 score = 99.7% Accuracy = 99.4% AUC = 95.1% **Furcation defects** Sensitivity = 89.2% Precision = 93.3% F1 score = 91.2% Accuracy = 83.7% AUC = 0.868	(+) Effective	The system showed the highest diagnostic performance in the detection of total alveolar bone losses and the lowest in the detection of vertical bone losses.	AI systems offer promising results in determining periodontal bone loss patterns and furcation defects from dental radiographs.
2	Lee C T et al. [39]	2022	Retrospective study	Deep CNN (DL-based CAD models)	To measure RBL to aid diagnosis	693 periapical radiographs (original dataset); 644 additional periapical images [RBL] (additional dataset)	Assessment of alveolar bone level	Intraoral digital radiographs	Independent examiners (periodontist and periodontal resident)	DSC for segmentation: over 0.91. Accuracy = 85%	(+) Effective	The proposed DL model provides reliable RBL measurements and image-based periodontal diagnosis.	This model has to be further optimized and validated by a larger number of images to facilitate its application.
3	Alotaibi G et al. [40]	2022	Retrospective study	Combination of deep CNN (VGG-16) and self-trained network	To detect and evaluate severity of bone loss due to periodontal disease	1724 periapical radiographs from 1610 adult patients [70% training, 20% validation, and 10% testing datasets]	Assessment of alveolar bone loss	Intraoral periapical images/radiographs	Three independent examiners, including a periodontist	Diagnostic accuracy for classifying normal versus disease was 73% and 59% for classification of the levels of severity of the bone loss. Precision, recall, and F1 scores for the binary classifier were above 70%.	(+) Effective	The deep CNN (VGG-16) was useful to detect alveolar bone loss as well as to detect the severity of bone loss in teeth.	A computer-aided detection system should be able to aid in the detection and staging of periodontitis.
4	Chang HJ et al. [41]	2020	Observational study	[Hybrid framework] Combined CNN (Mask R-CNN) and conventional CAD approach	To automatically detect and classify the periodontal bone loss of each individual tooth	330, 115, and 73 panoramic radiographs (90% training set and 10% test set)	Assessment of alveolar bone loss	DPRs	Radiologists (professor, fellow, and residents)	Pearson correlation = 0.73, and the intraclass correlation value = 0.91 overall for the whole jaw	(+) Effective	The novel hybrid framework demonstrated high accuracy and excellent reliability in the automatic diagnosis of periodontal bone loss and the staging of periodontitis.	The framework may substantially improve dental professionals’ performance with regard to the diagnosis and treatment of periodontitis.
5	Kim J et al. [42]	2019	Retrospective study	Deep CNN (DeNTNet)	To detect PBL with teeth numbering	12,179 panoramic dental radiographs [11, 189 (trained), 190 (validated), and 800 (tested)]	Assessment of alveolar bone loss	DPRs	Experienced dental hygienists with 5, 9, 16, 17, and 19 years of practice	When compared to dental clinicians F1 score of 0.75 on the test set, the average performance of dental clinicians was 0.69	(+) Effective	This proposed model was able to achieve a PBL detection performance superior to that of dental clinicians.	This approach substantially benefits clinical practice by improving the efficiency of diagnosing PBL and reducing the workload involved.
6	Chang J et al. [43]	2022	Retrospective study	Multitasking Inception V3 model (deep machine learning)	To test the accuracy of radiographic bone loss (RBL) classification	236 patients with 1836 periapical radiographs	Assessment of alveolar bone loss	Periapical digital radiographs	Three calibrated periodontists	Accuracy = 87% Sensitivity = 86% Specificity = 88% PPV = 88% NPV = 86%	(+) Effective	Application of deep machine learning for the detection of alveolar bone loss yielded promising results.	Higher accuracy of RBL classification can be achieved with more clinical data and proper model construction for valuable clinical application by machine learning.
7	Krois J et al. [44]	2019	Observational study	Deep CNN	To detect PBL on panoramic scans	85 randomly chosen radiographs	Assessment of alveolar bone loss	DPRs	Six experienced dentists	**CNN** Accuracy = 81% Sensitivity = 81% Specificity = 81% **Dentist** Accuracy = 76% Sensitivity = 92% Specificity = 63%	(+) Effective	A moderately complex trained CNN showed at least a similar diagnostic performance to that of experienced dentists.	Dentists’ diagnostic efforts when using radiographs may be reduced by applying machine learning-based technologies.
8	Danks RP et al. [45]	2021	Retrospective study	Deep neural network with hourglass architecture	To determine the disease severity stage and regressive percentage of PBL	340 fully anonymized periapical radiographs	Classification of periodontitis and assessment of PBL	Periapical radiographs	Postgraduate specialist trainees	The landmark localization achieved percentage correct key points of 88.9%, 73.9%, and 74.4%, respectively, and a combined PCK of 83.3%. When compared, the average PBL error was 10.69%, with a severity stage accuracy of 58%.	(+) Effective	The system showed the promising ability to localize landmarks and estimate periodontal bone loss.	Future work is required so that a computer-assisted radiographic assessment system can provide significant support in periodontitis and interventional application.
9	Kim SH et al. [46]	2022	Observational study	CNN models (U-Net, Dense-UNet, and U^2^-Net)	To measure quantitatively and automatically the alveolar bone level by detecting the CEJ junction and alveolar bone crest	500 images were scanned manually [400 images for training, 50 images for validation, and 50 images for testing]	Assessment of alveolar bone level	OCT images; optical coherence tomography	One periodontist with seven years of experience	All CNN models showed MAEs of less than 0.25 mm in the x and y coordinates and greater than 90% successful detection rates at 0.5 mm for both the ABC and CEJ	(+) Effective	The CNN models showed high segmentation accuracies in the tooth enamel and alveolar bone regions, as well as high correlation and reliability with ABL.	The proposed method has the potential to be utilized in periodontitis diagnosis or other clinical periodontal procedures.
10	Kabir T et al. [47]	2021	Observational study	Deep learning network HYNETS	To evaluate HYNETS in grading periodontitis and RBL assessment	700 X-rays were divided into training, testing, and validation sets	Assessment of alveolar bone loss	Periapical radiographic images	Periodontists (board-certified clinical and board-certified professor) and resident	HYNETS achieved average Dice coefficients of 0.96 and 0.94 for the bone area and tooth segmentation and an average AUC of 0.97 for periodontitis stage assignment.	(+) Effective	HYNETS could potentially transform clinical diagnosis from a manual, time-consuming, and error-prone task to efficient and automated periodontitis stage assignment.	HYNETS could be useful in the future for integration and will be successful in clinical practice.
11	Jiang L et al. [48]	2022	Retrospective study	Deep learning [U-Net and YOLO v4]	To establish a comprehensive and accurate radiographic staging of PBL	640 panoramic images	Assessment of alveolar bone loss	Panoramic images	Three experienced periodontal physicians	The overall classification accuracy of the model was 77%.	(+) Effective	The model classification was more accurate than that of general practitioners in detecting and classifying alveolar bone loss.	The model could assist dentists in the comprehensive and accurate assessment of PBL.
12	Uzun Saylan BC et al. [49]	2023	Observational study	PyTorch-based YOLO-v5 model	To evaluate the success of AI models used in the detection of radiographic alveolar bone loss	685 panoramic radiographs (80% training 10% validation, and 10% testing)	Assessment of alveolar bone level	DPRs	Oral and maxillofacial radiologist and periodontologist with at least 10 years of experience	ABL Sensitivity = 75% Precision = 76% F1 score = 76%	(+) Effective	The lowest sensitivity and F1 score values were associated with total alveolar bone loss, while the highest values were observed in the maxillary incisor region.	The study shows that artificial intelligence has high potential in analytical studies evaluating periodontal bone loss situations.
**Table 2d: Application of AI to predict periodontal diseases**													
**Sl No.**	**Authors**	**Year of Publication**	**Study Design**	**Algorithm** **Architecture**	**Objective of the Study**	**No. of Patients/Images/Photographs for Testing**	**Primary Objective**	**Modality**	**Comparison, If Any**	**Evaluation Accuracy/Average Accuracy/Statistical Significance**	**Results:** **(+) Effective,** **(−) Noneffective, (N) Neutral**	**Outcomes**	**Author Suggestions/Conclusions**
1	Shimpi N et al. [50]	2020	Cohort study	ANN DT	To propose and test a new PD risk assessment model	11,048 (4766 positive and 6282 controls)	Prediction of periodontal risk	Datasets	NB LR SVM	DT showed a sensitivity of 87.08% and a specificity of 93.5%; DT and ANN demonstrated higher accuracy in classifying patients with high or low PD risk as compared to NB, LR, and SVM.	(+) Effective	ML methods would be effective when applied to improving patient care through the early detection of PD or to new preventive approaches to PD by assisting healthcare professionals to evaluate patients’ PD risk.	Evaluation of performances of these algorithms in other populations is essential to demonstrate their generalizability and relevance and utility as clinical decision support tools in the medical setting.
2	Vadzyuk S et al. [51]	2021	Cross-sectional study	Neural networks	To predict the development of periodontal disease	156 students [84 people with periodontal disease and 72 without periodontal pathology (control)]	Prediction of periodontal disease	Datasets	Conventional methods and expert opinions	The diagnostic sensitivity of the first prognostic model was 83.33%, and the specificity was 92.31%. The second model was characterized by 90.00% sensitivity and 78.57% specificity.	(+) Effective	The psychophysiological features can be effective predictors of the development of pathologies and periodontal tissues including inflammation.	The method of modeling using neural networks can effectively predict the risk of periodontal disease development in young people.
3	Kearney VP et al. [52]	2022	Retrospective study	Inpainting network	To enhance the CAL prediction accuracy	80,326 images were used for training, 12,901 for validation, and 10,687 to compare CALs	Prediction of periodontal disease	Bitewing and periapical radiographs	Experienced academic practicing clinicians (certified periodontist, 11 years of experience, and two dentists, 22 and 38 years of experience)	Comparator *p*-values demonstrated statistically significant improvement in CAL prediction with MAEs of 1.04 mm and 1.50 mm.	(+) Effective	The use of a generative adversarial inpainting network with partial convolutions to predict CALs from bitewing and periapical images is superior.	Artificial intelligence was developed and utilized to predict clinical attachment levels compared to clinical measurements.
4	Li H et al. [53]	2021	Retrospective study	Mask R-CNN (deetal-Perio)	To predict the severity of periodontitis	First data: 302 digitized panoramic radiographs; second data: 204 panoramic radiographs	Prediction of severity of periodontitis	DPRs	Expert dentist with more than 10 years of experience	**First dataset:** Macro F1 score = 0.894 Accuracy = 89.6%, **Second dataset:** Macro F1 score = 0.820 Accuracy = 82.4%	(+) Effective	This system outperformed state-of-the-art methods and showed robustness on two datasets in periodontitis prediction.	Deetal-Perio is a suitable method for periodontitis screening and diagnostics.
5	Lee JH et al. [54]	2018	Retrospective study	deep CNN	To develop and evaluate the accuracy of the model for the diagnosis and prediction of PCT	1740 periapical radiographic images into training dataset (1044), validation dataset (348), and datasets (348) for molars and premolars	Diagnosis and prediction of periodontitis	Periapical radiographs	Three calibrated periodontists	Diagnostic accuracy for PCT was 81% for premolars and 76.7% for molars. Accuracy of prediction extraction for premolars was 82.8% and an AUC of 82.6% for deep CNN models.	(+) Effective	The deep CNN algorithm had higher diagnostic accuracy for identifying PCT among premolars than among molars. They had similar diagnostic and predictive accuracies to those obtained by periodontists with respect to the prediction of extraction.	The system is expected to become an effective and efficient method for diagnosing and predicting PCT.

Footnotes: ML—machine learning; ANNs—artificial neural networks; CNNconvolutional neural networks; MLP—multi-layer perception; DT—decision tree; SVM—support vector machine; MLP—C-index–concordance index; CBCT—Cone–Beam Computed Tomography; OCT—optical coherence tomography; LR—Logistic Regression; SVM—Support Vector Machine; CLAHE + GLCM + ELM—contrast-limited adaptive histogram equalization, gray-level co-occurrence matrix, and extreme learning machine; DSC—Dice similarity coefficient; DPRs—dental panoramic radiographs; AgP—aggressive periodontitis; CP—chronic periodontitis; LM—Levenberg–Marquardet; SCG—scaled conjugate gradient; RBL—radiographic alveolar bone level; ABL—alveolar bone loss; MGLCM—multichannel gray-level co-occurrence matrix; PSONN—particle swarm optimization neural network.

**Table 3:** Assessment of strength of evidence.

Outcome	Inconsistency	Indirectness	Imprecision	Risk of Bias	Publication Bias	Strength of Evidence
Application of AI for diagnosing, classifying, and grading the severity of periodontal diseases [22,23,24,25,26,27,28,29,30,31,32,33]	Not Present	Not Present	Not Present	Present	Not Present	⨁⨁⨁◯
Application of AI to diagnose gingivitis [34,35,36,37]	Not Present	Not Present	Not Present	Present	Not Present	⨁⨁⨁⨁
Application of AI to evaluate radiographic alveolar bone level and severity of alveolar bone loss [38,39,40,41,42,43,44,45,46,47,48,49]	Not Present	Not Present	Not Present	Not Present	Not Present	⨁⨁⨁⨁
Application of AI to predict periodontal diseases [50,51,52,53,54]	Not Present	Not Present	Not Present	Present	Not Present	⨁⨁⨁⨁

⨁⨁⨁⨁—High evidence; ⨁⨁⨁◯—moderate evidence. The certainty of the studies included in this systematic review was evaluated using the Grading of Recommendations Assessment Development and Evaluation (GRADE) approach. Inconsistency, indirectness, imprecision, risk of bias, and publication bias were the five domains that determined the certainty of evidence and can be categorized as very low, low, moderate, or high evidence. The overall certainty of evidence from the included studies in this review was found to be high.

## Data Availability

No new data were created or analyzed in this study. Data sharing is not applicable to this article.

## Supplementary Materials

The following supporting information can be downloaded at https://www.mdpi.com/article/10.3390/diagnostics15243247/s1, Table S1: Risk of Bias assessment.
