# Supplementary material for: The Application and Performance of Artificial Intelligence (AI) Models in the Diagnosis, Classification, and Prediction of Periodontal Diseases: A Systematic Review
Source: Diagnostics (Basel). 2025 Dec 18;15(24):3247. doi: 10.3390/diagnostics15243247 (PMC12731622; doi:10.3390/diagnostics15243247)
Supplement: Supplementary file 1 [file diagnostics-15-03247-s001.zip › diagnostics-3965160-supplementary.pdf]

		RISK OF BIAS				APPLICABILITY CONCERNS		
Sl No	Authors	Patient Selectio n	Index Test	Reference Standard	Flow And Timing	Patient Selection	Index Test	Reference Standard
**A. Application of AI for Diagnosing, Classifying, and Grading the Severity of Periodontal Diseases**								
1	Ossowska A et al [22]	LOW	LOW	LOW	LOW	LOW	LOW	LOW
2	Thanathornwong B et al [23]	LOW	LOW	LOW	LOW	LOW	LOW	LOW
3	Ozden F O et al [24]	LOW	LOW	LOW	LOW	LOW	LOW	LOW
4	Papantonopoulos G et al[25]	LOW	LOW	LOW	LOW	LOW	LOW	LOW
5	Xiang J et al[26]	LOW	HIGH	HIGH	HIGH	LOW	HIGH	HIGH
6	Farhadian M et al [27]	LOW	HIGH	HIGH	HIGH	LOW	HIGH	HIGH
7	Chifor R et al [28]	LOW	LOW	HIGH	LOW	LOW	LOW	HIGH
8	Arbabi S et al[29]	LOW	LOW	LOW	LOW	LOW	LOW	LOW
9	Su S et al [30]	LOW	LOW	LOW	LOW	LOW	LOW	LOW
10	Patel JS et al[31]	LOW	LOW	LOW	LOW	LOW	LOW	LOW
11	Shon, H.S. et al[32]	LOW	LOW	LOW	LOW	LOW	LOW	LOW
12	İçöz D et al [33]	LOW	LOW	LOW	LOW	LOW	LOW	LOW
**b: Application of AI to diagnose gingivitis**								
1	Alalharith D.M et al [34]	LOW	LOW	LOW	LOW	LOW	LOW	LOW
2	Li W et al[35]	LOW	LOW	LOW	LOW	LOW	LOW	LOW
3	Li W et al [36]	LOW	LOW	LOW	LOW	LOW	LOW	LOW
4	Li W et al[37]	LOW	LOW	LOW	LOW	LOW	LOW	LOW
**c: Application of AI to evaluate radiographic alveolar bone level and severity of alveolar bone loss**								
1	Kurt-Bayrakdar S et al [38]	LOW	LOW	LOW	LOW	LOW	LOW	LOW
2	Lee C T et al [39]	LOW	LOW	LOW	LOW	LOW	LOW	LOW
3	Alotaibi G et al [40]	LOW	LOW	LOW	LOW	LOW	LOW	LOW
4	Chang HJ et al [41]	LOW	LOW	LOW	LOW	LOW	LOW	LOW
5	Kim J et al [42]	LOW	LOW	LOW	LOW	LOW	LOW	LOW
6	Chang J et al[43]	LOW	LOW	LOW	LOW	LOW	LOW	LOW
7	Krois J et al [44]	LOW	LOW	LOW	LOW	LOW	LOW	LOW
8	Danks RP et al[45]	LOW	LOW	LOW	LOW	LOW	LOW	LOW
9	Kim SH et al[46]	LOW	LOW	LOW	LOW	LOW	LOW	LOW
10	Kabir T et al[47]	LOW	LOW	LOW	LOW	LOW	LOW	LOW
11	Jiang L et al [48]	LOW	LOW	LOW	LOW	LOW	LOW	LOW
12	Uzun Saylan BC et al. [49]	LOW	LOW	LOW	LOW	LOW	LOW	LOW
**d: Application of AI to predict periodontal diseases**								
1	Shimpi N et a l[50]	LOW	LOW	LOW	LOW	LOW	LOW	LOW
2	Vadzyuk S et al[51]	LOW	LOW	LOW	LOW	LOW	LOW	LOW
3	Kearney VP et al [52]	LOW	LOW	LOW	LOW	LOW	LOW	LOW
4	Li H et al[53]	LOW	LOW	LOW	LOW	LOW	LOW	LOW
5	Lee JH et al [54]	LOW	LOW	LOW	LOW	LOW	LOW	LOW

Table S1: Risk of Bias Assessment
